# Comparison of the safety and efficacy of remimazolam *versus* propofol for sedation in patients undergoing fiberoptic bronchoscopy with preserved spontaneous breathing: a retrospective analysis

**DOI:** 10.3389/fphar.2026.1796709

**Published:** 2026-04-24

**Authors:** Junjie Song, Tianyi Ge, Jing Liu, Weiwei Chen, Jiahang Chen, Kexin xie, Ying Wang

**Affiliations:** The First Affiliated Hospital of Henan University, Henan University, Kaifeng, China

**Keywords:** adverse events, fiberoptic bronchoscopy, propofol, remimazolam, satisfaction

## Abstract

**Background and Objectives:**

Adequate sedation with preserved spontaneous breathing and stable hemodynamics is critical for the success and safety of fiberoptic bronchoscopy (FOB). Remimazolam, a novel ultra-short-acting benzodiazepine with rapid metabolism and favorable sedative profiles, has shown promising sedative effects for procedural sedation. However, comparative data on its application in FOB with preserved spontaneous breathing remain limited. This retrospective study aimed to compare the safety and efficacy of remimazolam and propofol for sedation in patients undergoing FOB with preserved spontaneous breathing.

**Materials and Methods:**

A retrospective chart review was conducted from October 2024 to October 2025. All patients undergoing FOB with preserved spontaneous breathing were enrolled. Patients were divided into two groups based on the sedative used: remimazolam (Group R) and propofol (Group P). The primary outcome was the sedation success rate. Secondary outcomes included hemodynamic parameters, incidence of adverse events, anesthesia-related times, satisfaction scores of endoscopists and patients.

**Results:**

Thirty-six patients in group R and Forty in group P were collected in this retrospective study. Sedation success rate was 100% in both groups. Oxygen saturation (SpO_2_) levels were higher in group R at beginning of the bronchoscopy, 5, 10, and 15 min after the start of bronchoscopy (*P* = 0.012, 0.001, 0.017, and 0.006, respectively). Group R had a lower incidence of hypoxemia (16.67% vs*.* 35.0%, *P* = 0.034), injection pain (0% vs*.* 17.5%, *P* = 0.008), and hypotension (5.56% vs*.* 22.5%, *P* = 0.036). Recovery time was shorter in group R (*P* = 0.017). Onset time, and satisfaction scores were comparable in the two groups.

**Conclusion:**

Our study uncovered remimazolam demonstrates a comparable success rate to propofol and exhibits favorable safety profiles, including reduced hypoxemia, injection pain, and hypotension during FOB with preserved spontaneous breathing. However, the shorter recovery time in Group R was confounded by the routine use of flumazenil, and the findings are limited by the retrospective design, small sample size and exclusive enrollment of ASA I–II patients, requiring validation in larger, prospective studies.

## 1 Introduction

Fiberoptic bronchoscopy (FOB) is an important method for diagnosing and treating respiratory diseases in clinical practice. However, during implementation, the mechanical stimulation of a bronchoscope entering the airway can cause transient airway obstruction and increase airway resistance. Concurrently, the rich nerve distribution in the nose, pharynx, and throat can trigger strong stress responses, which manifest as increased heart rate, elevated blood pressure, severe cough, nausea, and vomiting and may induce serious complications such as hemoptysis and asthma (Zhang et al., 2023). To attenuate these responses, relieve anxiety and pain, and improve patient cooperation, moderate-to-deep sedation is usually adopted without affecting hemodynamics and oxygenation (Chen et al., 2022). Therefore, a safe and controllable sedation/analgesia protocol that minimally interferes with the respiratory and circulatory systems is crucial for FOB.

A major challenge during painless FOB is achieving sufficient depth of sedation while maintaining spontaneous ventilation, hemodynamic stability, and oxygenation. The selection of a sedation regimen directly influences both procedural efficacy and patient experience. Remimazolam and propofol are commonly used anesthetic drugs during FOB. Propofol has the advantages of rapid anesthesia onset, short action duration, and quick anesthesia recovery (Gaisl et al., 2016; Xing et al., 2024) and is the most widely used anesthetic drug in bronchoscopy. However, propofol has several limitations, such as dose-dependent respiratory and cardiovascular depression (Chang et al., 2023; Chen W. et al., 2024), a narrow therapeutic window, lack of a specific antagonist, and injection site pain (Zhang et al., 2021). In addition, the applicability of propofol is limited in patients with egg and/or soybean allergy (Chen S. et al., 2020).

Remimazolam is a novel ultra-short-acting benzodiazepine that acts as a selective agonist of gamma-aminobutyric acid type A (GABAA) receptors (Kilpatrick et al., 2007; Keam, 2020; Chen Q. et al., 2024). Remimazolam has the advantages of rapid onset, quick recovery, and strong controllability and has a lower impact on the circulatory and respiratory systems than propofol (Wang et al., 2022; Lee et al., 2023), with few side effects such as injection site pain (Koo et al., 2025). The onset time of remimazolam is 1–3 min, and its metabolic half-life is approximately 0.75 h. It has a rapid onset–offset profile comparable to that of propofol. Remimazolam is metabolized by nonspecific tissue esterases, independent of hepatic or renal function (Saari et al., 2011). In addition, its effects can be completely reversed by flumazenil (Kilpatrick et al., 2007; Worthington et al., 2013; Chen X. et al., 2020). These pharmacological characteristics position remimazolam as a potentially ideal agent for painless endoscopic procedures.

Although several studies have compared the effects of remimazolam and propofol, most have focused only on related without maintaining spontaneous breathing (Pan et al., 2022; Chai et al., 2025; Hua et al., 2025) or were conducted in non-bronchoscopy procedures (Pedersen et al., 2023). Till now, there is no standardized pharmacological sedative suggested for bronchoscopy. Two sedative options are commonly used in our hospital for sedation of FOB, including propofol and remimazolam. Therefore, the purpose of our retrospective study was to investigate the efficacy and safety of remimazolam *versus* propofol during FOB with preserved spontaneous breathing.

## 2 Materials and methods

**FIGURE 1:**
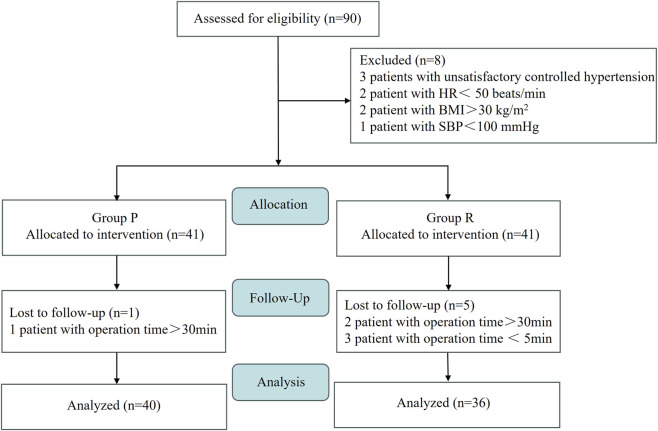
Flowchart of the current trial.

This retrospective study was approved by the Ethics Committee of the First Affiliated Hospital of Henan University (Approval Number: 2025-03-142). All methods were carried out in compliance with the Declaration of Helsinki and other relevant national and international guidelines and regulations for medical research involving human participants. A total of 90 patients who underwent FOB with preserved spontaneous breathing from October 2024 to October 2025 were selected (Figure 1).

### 2.1 Study population

We included elective bronchoscopic procedures performed under total intravenous anesthesia. We reviewed anesthesia records of bronchoscopy and further divided the cases into two groups based on sedatives, either propofol (group P) or remimazolam (group R).

The inclusion criteria were as follows: age between 18 and 75 years, an American Society of Anesthesiologists (ASA) physical status of I–II, a body mass index (BMI) of 18–30 kg/m^2^, and diagnosis and/or treatment through bronchoscopy.

The exclusion criteria were as follows: refusal to participate; previous mental or psychological disorders; a difficult airway (Mallampati score ≥ III); long-term use of anesthetic analgesics, sedatives, or nonsteroidal anti-inflammatory drugs; known allergy to remimazolam, propofol, or similar drugs; a preoperative heart rate (HR) of <50 times/min; a systolic blood pressure (SBP) of <100 mmHg; severe hepatic dysfunction (Child–Pugh class C); severe renal dysfunction (requiring dialysis); a BMI of >30 kg/m^2^; and operating time >30 min or <5 min.

### 2.2 Anesthesia protocol

None of the patients received any medication before anesthesia induction. Pre-oxygenation was performed with 100% oxygen for 5 min. Group R was administered 0.2 mg/kg remimazolam (Yichang Ren fu Pharmaceutical Co., Ltd., batch number: AC5030091, China) intravenously. Group P was administered 2.0 mg/kg propofol (Chenxin Pharmaceutical Co., Ltd., batch number: D24123071, China) intravenously. Subsequently, both groups were administered 0.2 μg/kg sufentanil (Yichang Ren fu Pharmaceutical Co., Ltd., lot number: AB41100411, China) and 0.2 mg glycopyrrolate (Yuandong Biopharma Co., Ltd., lot number: 240403, China) for anesthesia induction. When the Modified Observer’s Assessment of Alertness/Sedation (MOAA/S) score was ≤3, a laryngeal mask was inserted. All patients retained spontaneous breathing and received oxygen through a nasal cannula at a rate of 5 L/min. During the operation, sedation was maintained, and one-third of the initial dose of remimazolam (group R) or propofol (group P) was administered to maintain the MOAA/S score ≤1 to maintain an appropriate level of anesthesia. An additional dose equivalent to one-third of the initial induction dose was administered when the MOAA/S score ≥ 2, or when patients presented with body movement, cough, which indicated insufficient sedation depth. The definition of sedation failure (≥5 supplementary doses within 15 min) was established based on institutional clinical practice for FOB sedation, where repeated bolus administration beyond this threshold indicates inadequate initial sedation and poor clinical controllability. In group R, 0.2 mg flumazenil (Yichang Ren fu Pharmaceutical Co., Ltd., batch number: AB40505521, China) was intravenously injected immediately after the operation. When the Steward score was ≥4, the laryngeal mask was removed. All patients were continuously monitored for at least 30 min in the post-anesthesia care unit (PACU) to observe the occurrence of re-sedation, which was defined as a decrease in MOAA/S score to <4 after initial recovery to ≥4, or requiring additional flumazenil administration.

### 2.3 Data Collection

All data were obtained from anesthesia documents within the chart and analyzed by an investigator blinded to our study. Patient characteristics included demographic data, ASA physical status, disease diagnosis, bronchoscopic procedure, anesthesia and procedure time. The primary outcome was the success rate of sedation. Secondary outcomes were HR; SBP; diastolic blood pressure (DBP); and SpO_2_ at T0 (before induction), T1 (beginning of the bronchoscopy), T2 (5 min after the start of bronchoscopy), T3 (10 min after the start of bronchoscopy), T4 (15 min after the start of bronchoscopy), T5 (immediately after the ≧end of bronchoscopy), T6 (on awakening), and T7 (5 min after awakening).

Evaluation indices included anesthesia onset time (defined as the period from the start of intravenous administration until the MOAA/S score reached ≤ 3), anesthesia recovery time (defined as the period from the end of bronchoscopy until the MOAA/S score reached 5), hypoxemia (SpO_2_ < 90%), hypotension (SBP < 90 mmHg or <20% reduction in SBP from baseline), hypertension (SBP > 140 mmHg or >20% increase in SBP from baseline), bradycardia (HR < 50 beats/min), tachycardia (HR >100 beats/min), injection site pain, cough, nausea/vomiting, and body movement. Satisfaction scores of patients and bronchoscopists (0–3 points for dissatisfaction, 4–7 points for relative satisfaction, and 8–10 points for satisfaction) were also recorded.

### 2.4 Statistical analysis

Incidence of hypoxemia was selected to calculate the sample size. Initially, a total of 20 patients were included (10 in each group). The incidence of hypoxemia in the remimazolam group was 10%, while that in the propofol group was 40%. A power analysis was performed to determine the minimum sample size, which was calculated as 29 patients per group with a power of 0.8 and a type −1 error of 0.05. Considering a 15% shedding rate, 68 cases with 34 in each group were proposed for the study to meet statistical significance.

All statistical analyses were performed using the SPSS software (version 27.0). The Shapiro–Wilk and Levene tests were used to assess the distribution and homogeneity of the data. Normally distributed measurement data were expressed as the mean ± standard deviation and compared between groups using the independent-sample t-test. Date was reported as median if not normally distributed. Count data were expressed as numbers (percentages) or at a 95% confidence interval (CI) and compared between groups using the chi-square test. For all tests, a *P*-value < 0.05 was considered statistically significant.

## 3 Results

### 3.1 General data analysis of patients

We reviewed 90 consecutive patients undergoing FOB over a 12-month duration. Eight cases failed to meet the criteria, and six cases were excluded owing to meet the exclusion criteria. Documentation of 40 and 36 patients in the group P and group R were successfully analyzed, respectively. Patient characteristics of both groups are shown in Table 1. Both groups completed FOB according to the study protocol. The sedation success rate was 100% in both groups, with no significant difference. The general data of the patients and procedural details were significantly similar between the two groups (*P* > 0.05) (Table 1).

**TABLE 1:** Comparison of the patient baseline characteristics and procedural characteristic.

Variable	Group P (n = 40)	Group R (n = 36)	*P value*
Age (years)	62.85 ± 10.58	59.00 ± 14.12	0.18
Gender (n, %)	​	​	0.864
Male	23 (57.5%)	20 (55.5%)	​
Female	17 (42.5%)	16 (44.5%)	​
Height (cm)	166.98 ± 6.74	167.53 ± 8.92	0.061
Weight (kg)	68.65 ± 12.353	67.91 ± 12.34	0.979
Body mass index (kg/m^2^)	24.56 ± 3.71	24.16 ± 3.37	0.617
ASA I/II (n)	11/29	10/26	0.978
Comorbidity n (%)	​	​	0.954
Hypertension	10 (23.8%)	12 (33.33%)	​
Coronary heart disease	2 (4.8%)	4 (11.11%)	​
Diabetes	5 (11.9%)	4 (11.11%)	​
Chronic bronchitis/emphysema	4 (9.5%)	2 (5.6%)	​
Others	4 (9.5%)	2 (5.6%)	​
Indications for bronchoscopy n (%)	​	​	0.817
Lung cancer	14 (33.33%)	14 (38.9%)	​
Pneumonia	11 (26.2%)	8 (22.2%)	​
Bronchitis	1 (2.4%)	2 (5.6%)	​
Cough/Hemoptysis	13 (3.1%)	10 (27.8%)	​
Interstitial lung disease	3 (7.1%)	2 (5.6%)	​
Types of bronchoscopy n (%)	​	​	0.669
Check the airways	8 (19.0%)	6 (16.7%)	​
Biopsy	14 (33.33%)	12 (33.3%)	​
Bronchoalveolar lavage	20 (47.6%)	18 (50%)	​
Duration of bronchoscopy (min)	15.45 ± 8.70	16.77 ± 8.45	0.968
Duration of anesthesia (min)	19.86 ± 8.65	22.24 ± 9.35	0.701
Successfully sedation, n (%)	40 (100%)	36 (100%)	-

### 3.2 Hemodynamic variations during anesthesia

**FIGURE 2:**
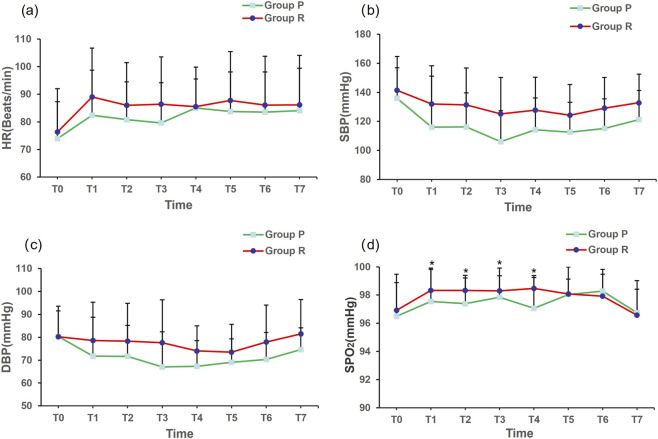
Hemodynamic variations in heart rate **(a)**, systolic blood pressure **(b)**, diastolic blood pressure **(c)** and pulse oximetry saturation **(d)**. Abbreviations: HR, heart rate; SBP, systolic blood pressure; DBP, diastolic blood pressure; SPO_2_, pulse oximetry saturation. *P value < 0.05 between two groups.

SpO_2_ levels were higher in group R than in group P at T1, T2, T3, and T4 (*P* = 0.012, 0.001, 0.017, and 0.006, respectively). No significant differences were found in HR, SBP, and DBP between the two groups at any time point (Figure 2).

### 3.3 Adverse events

The incidence rates of injection pain (0% vs*.* 17.5%, 95% CI: 0.715 to 0.952, *P* = 0.008), hypoxemia (16.67% vs*.* 35%, 95% CI: 0.095 to 0.943, *P* = 0.034), and hypotension (5.56% vs*.* 22.5%, 95% CI: 0.041 to 1.011, *P* = 0.036) were lower in group R than in group P. No significant differences were found in the incidence rates of cough, body movement, tachycardia, and hypertension between the two groups. Bradycardia was not observed in either group (Table 2).

**TABLE 2:** Incidence of intraoperative adverse events.

Variable (n, %)	Group P (n = 40)	Group R (n = 36)	*P* value
Injection pain	7 (17.5%)	0 (0%)	0.008
Cough	8 (20%)	9 (25.00%)	0.601
Body movement	7 (17.5%)	10 (27.78)	0.283
Tachycardia	0 (0)	1 (2.78%)	0.289
Bradycardia	0 (0)	0 (0)	-
Hypoxemia	14 (35%)	5 (16.67%)	0.034
Hypotension	9 (22.5%)	2 (5.56%)	0.036
Hypertension	0 (0)	1 (2.78%)	0.289

### 3.4 Sedative effects and satisfaction between the two groups

No significant difference was found in the onset time of anesthesia between the two groups (*P* > 0.05). However, recovery time of anesthesia was shorter in group R than in group P (3.58 [5.53 to 8.08] min vs*.* 6.36 [5.14 to 9.68] min, *P* = 0.017). The satisfaction scores of patients and bronchoscopists were similar between the two groups (*P* > 0.05) (Table 3).

**TABLE 3:** Sedative effects and satisfaction scores between the two groups.

Variable	Group P (n = 40)	Group R (n = 36)	*P* value
Onset time(min)	2.99 ± 1.01	2.74 ± 0.57	0.183
Recovery time (min)	6.36 [5.14 to 9.68]	3.58 [5.53 to 8.08]	0.017
Patient satisfaction	9.35 ± 0.622	9.67 ± 0.586	0.302
Physician satisfaction	9.08 ± 0.694	9.36 ± 0.762	0.082

## 4 Discussion

According to the guidelines established by the American College of Chest Physicians and the British Thoracic Society, measures of surface anesthesia, analgesia, or sedation should be used for all patients undergoing bronchoscopy in the absence of contraindications (Wahidi et al., 2011; Du Rand et al., 2013). However, maintaining a stable SpO_2_ level is a major challenge in painless FOB because anesthesiologists and endoscopists need to share the same airway, and the interactions and synergistic effects between sedatives and opioids can significantly increase respiratory depression (Finlay and Leslie, 2021). In this study, we compared the efficacy and safety of propofol and remimazolam in painless FOB with preserved spontaneous breathing. We found that both remimazolam and propofol provided satisfactory sedation and enabled complete examinations. In group R, the time to awakening was shorter, and no injection site pain occurred. No significant differences were found in hemodynamics, anesthesia onset time, satisfaction scores of patients and bronchoscopists, and incidence rates of some adverse events between groups R and P.

Chae et al. (Chae et al., 2022) reported that when a single intravenous injection of remimazolam was used for anesthesia induction, the effective dose 50 (ED50) and effective dose 95 (ED95) of remimazolam were 0.14 and 0.27 mg/kg, respectively. In a recent study on a Chinese population, the ED95 of remimazolam was found to be 0.219 mg/kg (Chen W. et al., 2021). Based on these results, a remimazolam concentration of 0.2 mg/kg was used for anesthesia induction in this study. Respiratory depression is a common adverse event of intravenous anesthetics. Both propofol and remimazolam can cause respiratory depression to a certain extent; however, the degree varies. In this study, SpO_2_ levels were higher in group R than in group P at T1, T2, T3, and T4, which may be related to the relatively mild inhibitory effects of remimazolam on respiration. In addition, the incidence of hypoxemia was lower in group R than in group P. These findings are consistent with those of a previous study (Xin et al., 2022). Remimazolam has high efficacy in maintaining the stability of respiratory and tidal volumes even at higher doses (Lohmer et al., 2020; Lu D. et al., 2024). These characteristics of remimazolam are particularly important in endoscopic procedures that require prolonged sedation, as they can ensure a relatively stable SpO_2_ level. Propofol, a potent intravenous anesthetic, has been widely reported to have dose-dependent respiratory depressant effects. It can significantly reduce respiratory rate and tidal volume and is more likely to cause respiratory depression or acute apnea (Jiang et al., 2021; Huang et al., 2022; Zhang W. et al., 2024).

Maintaining stable circulation is one of the core aspects of anesthesia in bronchoscopy. Although the HR, SBP and DBP in group R were higher than those in group P. No significant differences were found in HR, SBP and DBP between groups R and P at each time point. This reason may be related to the relatively mild inhibitory effects of remimazolam on the cardiovascular system. Propofol can decrease blood pressure and HR by exerting inhibitory effects on the peripheral vasculature and the heart in a dose-dependent manner, especially in elderly individuals or patients with cardiovascular disease (Kawasaki et al., 2018; Wu et al., 2024). On the contrary, remimazolam causes less interference with hemodynamics, maintains more stable circulation (Zhao et al., 2023; Shi et al., 2024), and is associated with a lower incidence of adverse events (Shi et al., 2024). This hemodynamic stability reduces not only the incidence of intraoperative hypotension and bradycardia but also the risk of postoperative cardiovascular complications. For younger individuals or those with good cardiac and pulmonary function, propofol and remimazolam can be used as effective drugs for sedation. However, for elderly patients or those with limited cardiac and pulmonary function, remimazolam has more clinical advantages owing to its relatively high level of safety.

In this retrospective trial, the incidence of injection site pain was higher in group P than in group R (*P* = 0.008). A notable finding in this study is the 0% incidence of injection site pain in the remimazolam group**,** which appears to contradict some previous literature reporting mild to injection pain associated with remimazolam (Lu L. et al., 2024). Similarly, Dai G et al.(Dai et al., 2021) also demonstrated that no pain was observed in remimazolam groups. Furthermore, research also confirmed that injection pain was not founded in the induction and maintenance of general anesthesia in surgical patients (Doi et al., 2020). However, injection pain in the remimazolam was observer during anesthesia induction, although the incidence was lower than that in the propofol (12.3% vs*.* 39.7%, *P* < 0.001) (Zhang L. et al., 2024). Larger, multicenter studies with more diverse patient populations and different remimazolam formulations may still detect cases of injection site pain (Choe et al., 2024; Xin et al., 2024; Zhou et al., 2024; Han et al., 2025), and clinicians should remain vigilant for this potential adverse event in clinical practice. While no injection site pain was observed in our study, this finding should be interpreted with caution. This discrepancy can be explained by two key factors: first, the formulation of remimazolam used in this study is a water-soluble tosylate salt with a neutral pH, which reduces local tissue irritation compared to the propofol emulsion (an oil-in-water formulation with acidic pH) that is well-known to cause injection pain. Second, the sample size of this study is small, and it is possible that the study was underpowered to detect mild injection pain. Third, this might be related to the analgesic drug sufentanil that we used in this study. Our study found that the incidence of cough and body movement was slightly higher in group R, it did not affect the progress of bronchoscopy and was not significantly different from that in group P. Similarly, a study on painless bronchoscopy in elderly patients indicated that compared with propofol, remimazolam had a lower incidence of adverse events and significantly reduced the risk of postoperative delirium (Shi et al., 2024). Compared to the propofol, we also noted that remimazolam decreases the incidence of hypoxia and hypotension. Clinically, hypoxemia is a critical adverse event during FOB with preserved spontaneous breathing, as it can lead to procedural interruptions, and increased perioperative risk. The 18.33% absolute reduction in hypoxemia incidence with remimazolam has important clinical value. The marked reduction in hypotension incidence has clear clinical implications. Hemodynamic stability is a core goal of FOB sedation, and reduced hypotension risk with remimazolam is particularly important for patients with cardiovascular diseases. Meta-analysis demonstrated that use of remimazolam for procedural sedation had lower incidence of hypoxia and hypotension compared to propofol (Zhang et al., 2022). Similarly, remimazolam was found associated with significantly lower rates of respiratory depression and hypotension for sedation in gastrointestinal endoscopic procedures (Barbosa et al., 2024).

This study shows that both propofol and remimazolam can achieve the sedation depth required for FOB. No significant differences were found in the success rate of sedation and the onset time of anesthesia between the two groups. In this study, sedation failure was defined as the need for more than five supplementary doses of sedative agent within any 15 min. Although this definition is not externally validated, it aligns with similar procedural sedation failure criteria reported in previous bronchoscopy study (Wu et al., 2022). Meanwhile, this was also based on our institution’s clinical practice for procedural sedation in FOB. In our practice, a high frequency of supplementary doses (≥5 doses within 15 min) indicates that the initial sedative bolus and titration strategy have failed to maintain adequate sedation depth, and this pattern of dosing was associated with an increased risk of over-sedation, adverse events, and procedural interruptions. However, anesthesia recovery time was significantly shorter in group R (*P* = 0.017). It should be noted that all patients in group R received flumazenil (0.2 mg) immediately after the procedure to reverse the sedative effects of remimazolam, whereas no antagonist was administered in group P. During monitoring in the recovery room, no event of re-sedation was observed in Group R after the administration of flumazenil. It is important to acknowledge that this difference is attributable to the use of flumazenil as a specific antagonist in group R. This is a key pharmacological feature of remimazolam—the ability to be rapidly and completely reversed by flumazenil (Wu et al., 2023). Therefore, we believe that the shortened recovery time observed in group R was due to the use of flumazenil and should not be construed as evidence that remimazolam has a faster recovery rate than propofol when there is no reversal effect. However, the magnitude of this natural recovery advantage cannot be accurately quantified in the context of this study due to the routine use of the antagonist. However, re-sedation of remimazolam after flumazenil antagonism has been reported in some cases (Yamamoto et al., 2021; Takemori et al., 2022). Musie’s study (Masui, 2023) indicated that the use of high doses of flumazenil (0.5 mg) in the presence of higher doses of remimazolam or in cases of low remimazolam clearance in the body increases the likelihood of re-sedation. This literature also noted that when the residual concentration of remimazolam is high, the reversal effect of flumazenil may disappear approximately 10 min after injection. In contrast, no re-sedation was observed in our study. Several factors may explain this difference. First, the operation times in our study were relatively short, with anesthesia durations averaging only about 20 min. Second, we used a lower dose of flumazenil (0.2 mg) for antagonism, which may have reduced the risk of re-sedation. Zhang’s study (Zhang L. et al., 2024) found time to eyes-opening, tracheal extubation was statistically shorter in the remimazolam group compared to propofol group in patients undergoing day surgery, but patients in the remimazolam group experienced re-sedation (10.8%) and hiccups (41.5%). We also noticed that in this study, the remimazolam group also administered flumazenil (0.5 mg). Recent studies have demonstrated that even without flumazenil reversal, remimazolam possesses favorable pharmacokinetic properties. Studies on gastroenterological endoscopy have shown that the time required to achieve full consciousness (3–10 min) is significantly shorter among patients sedated with remimazolam than among those sedated with propofol (Chen S. H. et al., 2021; Liu et al., 2021). Shorter recovery time may be related to the rapid hydrolysis of remimazolam by nonspecific tissue esterases in the blood into inactive metabolites (Kilpatrick et al., 2007). Consequently, accumulation of remimazolam is negligible even if it is administered continuously or at high doses (Tang et al., 2023). Furthermore, the use of flumazenil provides an additional safety advantage by enabling immediate and controllable recovery when needed and accelerate awakening (Worthington et al., 2013; An et al., 2025). In this study, all patients in group R were administered flumazenil after bronchoscopy, which may have led to rapid awakening. Thus, universal flumazenil administration in Group R represents a critical confounding factor for recovery time, and thus the shorter recovery time in Group R cannot be solely attributed to remimazolam’s intrinsic pharmacokinetic properties. On the contrary, propofol metabolism is affected by various factors, such as age, weight, and liver function (Guo et al., 2022).

This study has several limitations that should be considered. First, the design was not a randomized-controlled prospective study, the retrospective observational design inherently limits control over potential confounding factors (e.g., clinician preference for sedative agents, subtle differences in perioperative management), which may introduce bias in outcome comparisons. Second, the sample size was relatively small, small sample size (n = 76 analyzed) reduces statistical power to detect small but clinically meaningful differences in adverse events (e.g., bradycardia, hypertension) and limits the generalizability of the results. Further randomized controlled and large-scale trials would be required to confirm these findings. Third, the sedation failure definition is an institutional clinical practice criterion and has not been externally validated in large-scale trials, which may limit the comparability of sedation efficacy outcomes with other studies. Fourth, the routine use of flumazenil in Group R confounds the interpretation of recovery time, as discussed above, and no data on natural recovery from remimazolam sedation were collected in this study. Finally, all patients included in this study had ASA I–II without severe comorbidities, elderly patients with frailty, or ASA III–IV patients, which significantly limits the external validity of the findings. Collectively, the retrospective design, small sample size, and restricted patient enrollment mean that the findings of this study are only applicable to ASA I–II adult patients (18–75 years old) with a BMI of 18–30 kg/m^2^, no severe comorbidities, and undergoing elective FOB with preserved spontaneous breathing in a single center. The conclusions cannot be generalized to frail elderly patients, ASA III–IV patients, patients with severe underlying organ dysfunction, or pediatric/geriatric FOB patients outside this age range. In addition, the shorter recovery time observed in Group R cannot be directly extrapolated to clinical settings where flumazenil is not routinely used for remimazolam sedation.

## 5 Conclusion

In conclusion, this retrospective study shows that the sedation success rate of remimazolam is comparable to that of propofol in ASA I–II patients undergoing painless FOB with preserved spontaneous breathing. Remimazolam was associated with significantly less respiratory and circulatory systems, a short anesthesia recovery time, and a low incidence of injection site pain in this select population. However, the shorter recovery time observed in the remimazolam group was confounded by the routine administration of flumazenil. Although this study has some limitations, in clinical practice, remimazolam remains a promising and safe alternative sedative for patients undergoing FOB with preserved spontaneous breathing.

## Data Availability

The datasets analyzed during the current study available from the corresponding author on reasonable request.
